# Effective Connectivity Identifies Divergent Cerebro-Cerebellar Network Organization in Schizophrenia

**DOI:** 10.1007/s12311-026-01983-7

**Published:** 2026-03-23

**Authors:** Kami Pearson, Katrina Aberizk, Cindy An, Grace Hodges, Theo G.M. van Erp, Vince D. Calhoun, Jessica A. Turner

**Affiliations:** 1 Department of Psychiatry and Behavioral Health, The Ohio State University Wexner Medical Center, Columbus, OH USA; 2 Neuroscience Graduate Program, The Ohio State University, Columbus, OH USA; 3 Department of Psychology, The Ohio State University, Columbus, OH USA; 4 Clinical Translational Neuroscience Laboratory, Department of Psychiatry and Human Behavior, University of California Irvine, Irvine, CA USA; 5 Center for the Neurobiology of Learning and Memory, University of California Irvine, 309 Qureshey Research Lab, Irvine, CA USA; 6 Tri-Institutional Center for Translational Research in Neuroimaging and Data Science (TReNDS), Georgia State University, Georgia Institute of Technology, Emory University, Atlanta, GA USA

## Abstract

Introduction functional impairments in schizophrenia may arise from disruptions in large-scale brain networks. Emerging evidence highlights the cerebellum’s role in cognitive and affective regulations, yet its directional influence remains poorly understood. This study examines effective connectivity (EC) within cortico-striato-cerebellar networks in schizophrenia and healthy adults. Methods resting-state functional magnetic resonance imaging data from the Centers of Biomedical Research Excellence (COBRE), including people with schizophrenia and healthy controls (n = 134), were used to analyze intrinsic activity and effective connectivity. Cerebellar clusters showing reduced amplitude of low frequency fluctuations (ALFF) and fractional ALFF in schizophrenia were mapped to the Buckner 17-network atlas to define regions of interest (ROIs). Along with prefrontal and striatal ROIs defined a priori, these served as nodes in Group Iterative Multiple Model Estimation (GIMME). In addition to the best-fitting model of the full sample, schizophrenia participants were stratified by symptomology according to the positive and negative syndrome scale. Results four cerebellar voxel clusters in the posterolateral and anteromedial regions showed lower ALFF/fALFF in schizophrenia compared to controls. GIMME revealed distinct intra-cerebellar and cerebello-prefrontal EC patterns in schizophrenia that were absent in controls, including novel directed paths involving the cerebellar control network representation, consistent with its emergence as a central hub. These patterns persisted across symptom-defined subgroups, suggesting core network reorganization. Conclusions findings support the cerebellum’s involvement in disrupted network dynamics in schizophrenia, particularly its directional influence over prefrontal targets. EC analyses uncovered cerebellar reorganization that may underlie affective deficits in schizophrenia, offering novel targets for circuit-level interventions.

**Supplementary Information:**

The online version contains supplementary material available at 10.1007/s12311-026-01983-7.

## Introduction

Schizophrenia is a chronic psychiatric disorder characterized by positive (e.g. delusions, hallucinations, and disorganized speech) and negative symptoms (e.g. avolition, blunted affect, and asociality), the latter of which are particularly challenging to treat and strongly associated with functional impairment [1–4]. Historically, research has focused on dopaminergic and cortico-striatal dysfunction. However, growing evidence implicates cerebro-cerebellar circuitry in the emergence and severity of these symptoms [5–7]. Despite this, the cerebellar contribution to the characteristic patterns of hyper- and hypoconnectivity in schizophrenia has received comparatively little attention [8–10].

Traditionally considered a sensorimotor structure, the cerebellum is now recognized as a contributor to affective and cognitive processes, with fronto-cerebellar and cerebellar-striatal circuits supporting social prediction, goal-directed behavior, and reward processing [5, 11]. Resting-state functional magnetic resonance imaging (rs-fMRI), which measures spontaneous blood-oxygenation level dependent (BOLD) signal fluctuations, has been central to revealing these large-scale cerebro-cerebellar networks. In particular, the dorsolateral prefrontal cortex (dlPFC) and striatum are key targets of cerebellar output and have been consistently implicated in the pathophysiology of schizophrenia, including altered connectivity and dopamine signaling [12–14]. Structural connectivity between the cerebellum and dlPFC has been demonstrated in both nonhuman primate tracer studies and human diffusion tractography [15]. Similar evidence supports cerebellar projections to the caudate, putamen, and nucleus accumbens, forming anatomical pathways for cerebellar influence on circuits relevant to motivation, reward, and executive function [16–18]. These regions are also implicated in domains frequently disrupted in schizophrenia and linked to negative symptoms, such as goal-directed behavior, working memory, and reward processing.

Within this framework, cerebellar dysfunction can precede and shape cortical dysconnectivity via reciprocal cerebro-cerebellar loops [19]. Seminal research has emphasized cerebellar involvement in schizophrenia-related circuitry [20, 21]. Recent longitudinal evidence links conversion to schizophrenia in clinically high risk patients with hyperconnectivity within the cerebello-thalamo-cortical circuit [22, 23], a phenomenon further supported by targeted evidence of dentate nucleus hyperconnectivity with the dlPFC [24]. Aberrant connectivity between the cerebellum and prefrontal or parietal regions has been linked to symptom severity [25]. However, prior studies rely predominantly on static measures of functional coupling, which index synchrony but not directional influences between brain regions. Hence, it remains unknown whether the cerebellum influences cortical circuits in schizophrenia and how they relate to symptom domains.

To address this limitation, a range of effective connectivity approaches have been developed to estimate directed influences among brain regions. Statistical methods such as Granger causality, vector autoregressive models, and transfer entropy infer directionality based on temporal precedence but do not explicitly model underlying neural mechanisms and can be sensitive to sampling and hemodynamic confounds [26]. Generative model-based approaches, including dynamic causal modeling and whole-brain dynamical systems models, aim to infer latent neuronal interactions that give rise to observed signals, but are computationally intensive and typically constrained to smaller networks or strong prior assumptions [27, 28]. Among these approaches, Group Iterative Multiple Model Estimation (GIMME) offers a dynamic view of network interactions over time while accommodating substantial inter-individual variability, a key feature of schizophrenia [11, 29, 30].

Complementing this, amplitude of low frequency fluctuations (ALFF) and fractional ALFF (fALFF) quantify intrinsic neural activity at rest [31]. In the present study, ALFF and fALFF were first used to identify cerebellar regions of interest (ROIs) showing abnormal spontaneous activity in individuals with schizophrenia relative to healthy controls. This data-driven approach was motivated by the lack of clear consensus in the field regarding which cerebellar subregions are most consistently implicated in schizophrenia, in contrast to the striatal and cortical ROIs, which were selected based on prior findings [32–35]. By combining empirically derived cerebellar identification with theory-driven cortical and striatal targets, this approach enabled a focused examination of how cerebellar dysregulation may propagate through broader networks implicated in schizophrenia.

GIMME generates effective connectivity maps by estimating three effects: autoregressive, contemporaneous, and time-lagged. Autoregressive effects capture a region’s self-predictive activity across time points, reflective of dynamic stability or fluctuation. Because temporal autocorrelation varies systematically across cortical, subcortical, and cerebellar regions, deviations from typical patterns can reveal functional disruptions [36–40]. Contemporaneous effects reflect instantaneous dependencies between regions and may indicate altered integration within and between networks [41, 42]. Lagged effects capture directional influence across regions over time, offering insight into causal propagation of activity [43–46]. Effective connectivity is well suited to modeling cerebellar contributions to cortical networks, given the feedforward anatomical projections from the cerebellum to prefrontal and striatal targets, while also allowing for the detection of altered or compensatory directional influences that may emerge in neuropsychiatric disease.

We hypothesized that individuals with schizophrenia show differences in effective connectivity patterns both in intra-cerebellar and cerebro-cerebellar circuits relative to healthy controls. Given prior findings linking cerebellar-prefrontal dysconnectivity to symptom severity and disease progression [24, 25], we specifically predicted weaker directed connectivity from the cerebellum to the dlPFC associated with greater symptom severity. No a priori predictions were made regarding directionality of intra-cerebellar interactions, as these nodes were identified empirically. Through this framework, this study investigates how cerebellar dysfunction may influence broader network-level disruptions characteristic of schizophrenia. By identifying how these networks differ in patients with more severe symptoms, this work may inform future models of illness progression and guide circuit-targeted therapeutic strategies.

## Materials and Methods

### Participants

Rs-fMRI data was obtained from the Centers of Biomedical Research Excellence (COBRE) Phase 1 dataset [47]. Schizophrenia participants (SZ; *n* = 55) and age-matched healthy controls (HC; *n* = 79) were included in the present study, following data curation as described below (see Table 1**)**.

**Table 1:** Demographic and Clinical Characteristics

Age, mean (SD)	Persons with Schizophrenia (*n* = 55)	Healthy Controls (*n* = 79)	Overall (*n* = 134)
	36.0 (13.3)	37.2 (11.8)	36.7 (12.4)
**Sex**,** n (%)**			
Male	46 (83.6%)	57 (72.2%)	103 (76.9%)
Female	9 (16.4%)	22 (27.8%)	31 (23.1%)
**Baseline PANSS Scores**			
Positive, mean (SD)	14.5 (4.7)		
Negative, mean (SD)	14.9 (5.5)		
Total, mean (SD)	57.6 (15.5)		

*SD* = standard deviation

### Psychological Assessment

Details of the study assessments are presented in the parent study [47]. The stability of symptoms and medication was evaluated by reviewing retrospective psychiatric records for each individual with schizophrenia to confirm no changes had occurred within three months prior to study referral. Psychotic symptom severity was assessed using the Positive and Negative Syndrome Scale (PANSS) [48] within a week prior to neuroimaging. Positive and negative symptom PANSS subscales were summed, with each subscale ranging from 7 to 49. We used a median split of symptom scores for both positive and negative symptoms to divide the SZ group into low and high symptom severity subgroups, reflecting relatively milder and more severe symptomatology within the sample.

### MRI/fMRI Acquisition

A Siemens 3T TIM Trio scanner was used for image acquisition. 5-echo multi-echo MPRAGE sequence was used to acquire T1-weighted images [TE (echo times) = 1.65, 3.5, 5.36, 7.22, 9.08 ms; TR (repetition time) = 2.53 s; TI (inversion time) = 1.2 s; 7° flip angle, number of excitations (NEX) = 1; slice thickness = 1 mm; field of view (FOV) = 256 mm; resolution = 256 × 256]. 150 whole brain volumes were collected as part of the rs-fMRI scan, during which participants were instructed to look at a fixation cross for 5 min [TR = 2000 ms; TE = 29 ms; flip angle = 75°; FOV = 204 mm; matrix size = 64 × 64; 33 slices; voxel size = 3.75 × 3.75 × 4.55 mm, ascending order]. The first image of each run was discarded to account for T1 equilibrium effects.

### Data analysis—HALFPipe Preprocessing

The Enhancing Neuroimaging Genetics through Meta-analysis (ENIGMA) Harmonized Analysis of Functional MRI pipeline (HALFpipe) v1.2.1 was used for both MRI preprocessing and quality control, with Singularity v3.5.3 as the container platform. Preprocessing of functional resting-state data included slice time correction (ascending/interleaved scanning order), grand mean scaling (10,000) spatial smoothing (full-width at half maximum (FWHM) = 6 mm), temporal filtering (high-pass Gaussian-weighted filter with a width of 125 s), coregistration, and motion correction. Independent component analysis-based automatic removal of motion artifacts (ICA-AROMA) confound removal was used for the extraction of atlas-based connectivity matrices and BOLD signal time series [49]. Distortion correction was omitted, consistent with common practices for datasets lacking field maps.

As part of the quality control measures, each subject was visually inspected for T1w skull stripping and segmentation, T1w spatial normalization, EPI signal-to-noise ratio, ICA-based artifact removal, and spatial normalization. Subjects whose scans failed any of these measures, or who had a mean framewise displacement (FD) > 0.5 mm, were excluded from further analysis

A HALFpipe-specific atlas, Schaefer combined, was used for parcellation of the rs-fMRI BOLD signal to the T2-weighted anatomical images [49]. This atlas includes 400 cortical network parcellations derived from the Yeo [50] 17-network atlas [51], as well as FreeSurfer for subcortical regions [52], and the Buckner 17-network cerebellar atlas [53]. The Buckner atlas is a cerebellar atlas in which the parcels were defined based on their co-activation to the cortical networks of the Yeo atlas [7, 53].

### Data analysis—ROI Definition and Selection

ALFF and fALFF analyses were first conducted to empirically identify cerebellar regions exhibiting abnormal intrinsic activity in individuals with schizophrenia relative to healthy controls. This allowed us to empirically localize cerebellar dysfunction in our sample without presupposing network involvement. Significant clusters were identified using a cerebellar MNI mask and subsequently mapped to the Buckner 17-network cerebellar atlas to assign network-based labels. This procedure resulted in the selection of four cerebellar subregions, which served as inputs for GIMME to model directional connectivity between the cerebellum, striatum, and prefrontal cortex [54].

### Data Analysis—ALFF/fALFF

ALFF and fALFF are both measures of spontaneous fluctuations in BOLD signal activity within a voxel [31, 55]. HALFpipe automatically utilizes the temporal filtering (frequency range 0.01–0.1 Hz) from the preprocessing step to calculate ALFF, and further determines fALFF by calculating the ratio of the power in the low frequency range to the full frequency range [31, 49]. A general linear model of SZ vs. HC was conducted to identify cerebellar subregions of interest using a cerebellar MNI mask [56]. Using SPM12 v12.7771 in Matlab v24.1, a multiple regression was run using group, gender, and age as covariates.

### Data Analysis—GIMME

GIMME (version 0.7.18) was used to generate effective connectivity maps using predetermined ROIs [54]. First, BOLD time series data were compiled from all participants and structured in R (version 4.4.2) using a concatenated long-form approach. The following regions served as input for the GIMME analysis: bilateral dlPFC, bilateral superior dlPFC, and bilateral striatal subregions (caudate, nucleus accumbens, and putamen), and four cerebellar subregions identified through the ALFF/fALFF analyses (Cerebellum networks 8, 9, 13, and 17 from the Buckner 17 cerebellar atlas). BOLD signals were spatially averaged within their respective cortical parcellations (**Table ****S1**), yielding a single representative time series per cortical ROI. This list was then provided to the GIMME function of the *gimme* package [57] along with a vector identifying diagnostic groups/subgroup defined as follows. One statistical model compared schizophrenia (SZ) and healthy control (HC) groups, and a second statistical model compared individual with schizophrenia with high and low positive or negative symptom severity. Because group membership was specified a priori, a supervised approach was used, where predefined group labels guided subgroup-level path estimation. Parameters estimated in the full sample are referred to as SZ + HC throughout this report, and models estimated in SZ alone defined by symptom severity are referred to as SZ-only models. The GIMME subgroup and standardize options were set to TRUE. GIMME first generates autoregressive paths for all individuals included in the model, followed by group-level paths which are paths shared by 75% of the entire sample. Finally, subgroup-level paths are estimated and represent paths relevant to 75% of participants in either subgroup, consistent with the current default threshold implemented in the GIMME package. Group- and subgroup-level paths include both contemporaneous and lagged estimates, with lagged paths constrained to a lag of 1 (i.e., temporal influence from time *t* – 1 to time *t*).

### Secondary Analyses

Secondary analyses included multiple regressions for diagnostic group/subgroup differences in all autoregressive, lagged, and contemporaneous group-level effective connectivity path coefficients generated in each GIMME model. The number of paths tested corresponds to the total number of group-level paths estimated by GIMME in each mode (SZ + HC: *26 paths*; negative symptom severity: *29 paths*; positive symptom severity: *29 paths*). Autoregressive estimates were tested for all brain regions selected for the GIMME analysis, whereas contemporaneous and lagged paths were only those that appeared relevant for that particular model. In the SZ + HC comparisons, age, sex, and mean framewise displacement were included as covariates. In addition, the SZ-only models (i.e. positive and negative symptom severity) two additional covariates, namely the illness-to-life ratio and Olanzapine equivalency scores, were included [58–60]. Illness-to-life ratio was calculated as the duration of illness divided by the participant’s age, providing a normalized measure of chronicity. **Supplemental Fig. ****3** illustrates the distribution of predicted values for cerebellum 13 to 8, cerebellum 8 to 9, and autoregressive estimates for the left putamen and right caudate by diagnostic group. We used a false discovery rate (FDR) of *p* < 0.05 to correct for multiple comparisons [61].

Additionally, static functional connectivity was assessed using connectivity matrices derived from the Schaefer combined atlas using the same ROIs as selected for the GIMME analysis for comparison. This analysis was conducted for comparison purposes, but it was not the central focus of the study. Detailed methods and results for this additional analysis are provided in the supplemental materials (**Supplemental Figs. ****1**** and ****2**).

## Results

### ALFF/fALFF

Four distinct cerebellar clusters of voxels were identified, each exhibiting lower ALFF/fALFF in SZ compared to HC (FDR-corrected *p* < 0.05). The peak coordinates of each cluster were localized using the Buckner 17-network cerebellar atlas to determine their functional correspondence. Detailed information for each cluster, including MNI coordinates, statistical significance, and the corresponding Buckner networks, is provided in Table 2. Although cerebellar network 13 did not survive FDR correction at the voxel level (*p* = 0.078 [fALFF] / 0.093 [ALFF]), clusters with peak coordinates corresponding to this network appeared repeatedly across both ALFF and fALFF analyses. As a result, this region, along with the significant cerebellar networks 8, 9, and 17, were included in subsequent GIMME analyses.

**Table 2:** Clusters showing significantly greater ALFF/fALFF in healthy controls compared to individuals with schizophrenia

Cluster	MNI Coordinates	Cluster size (K_E_)	Peak T	*p* (FDR)	Buckner Network	Anatomical Region	Cortical Network	Analysis
1	32–42 -40	30	4.11	0.015*	8	VI, VIIb, Crus I-II	Salience	ALFF
2	-28 -42 -36	34	4.14	0.020*	9	VIII, Vermis III	Limbic	fALFF
3	-14 -82 -24	20	5.29	0.078 0.093	13	Crus I, VIIb	Control	fALFF ALFF
4	32–72 -38	27	4.28	0.015*	17	Crus I-II	Default	ALFF

For each cluster, peak *MNI* coordinates, cluster size (K_E_), T-values, p-values, and corresponding Buckner 17-network cerebellar atlas labels are reported. Clusters identified from both *ALFF* and *fALFF* analyses are included. *All clusters pass *FDR* correction (*p* < 0.05), except for one subthreshold cluster (*p* = 0.078 [*fALFF*] / 0.093 [*ALFF*]) that appeared across both *ALFF* and *fALFF* analyses and was therefore retained for further analysis

### GIMME-Identified Paths

**Fig. 1:**
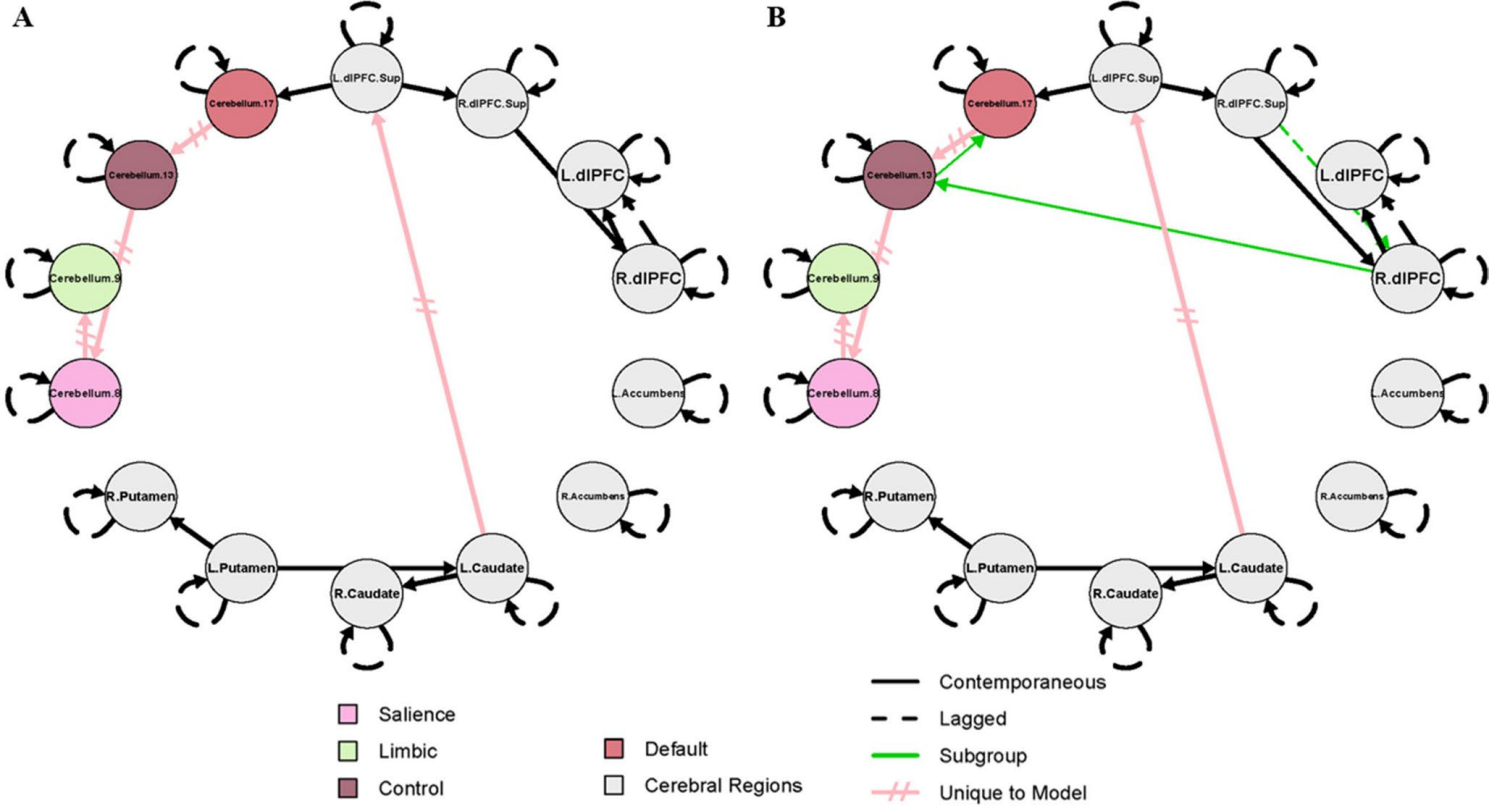
Effective connectivity maps for healthy controls and people with schizophrenia

**Fig. 2:**
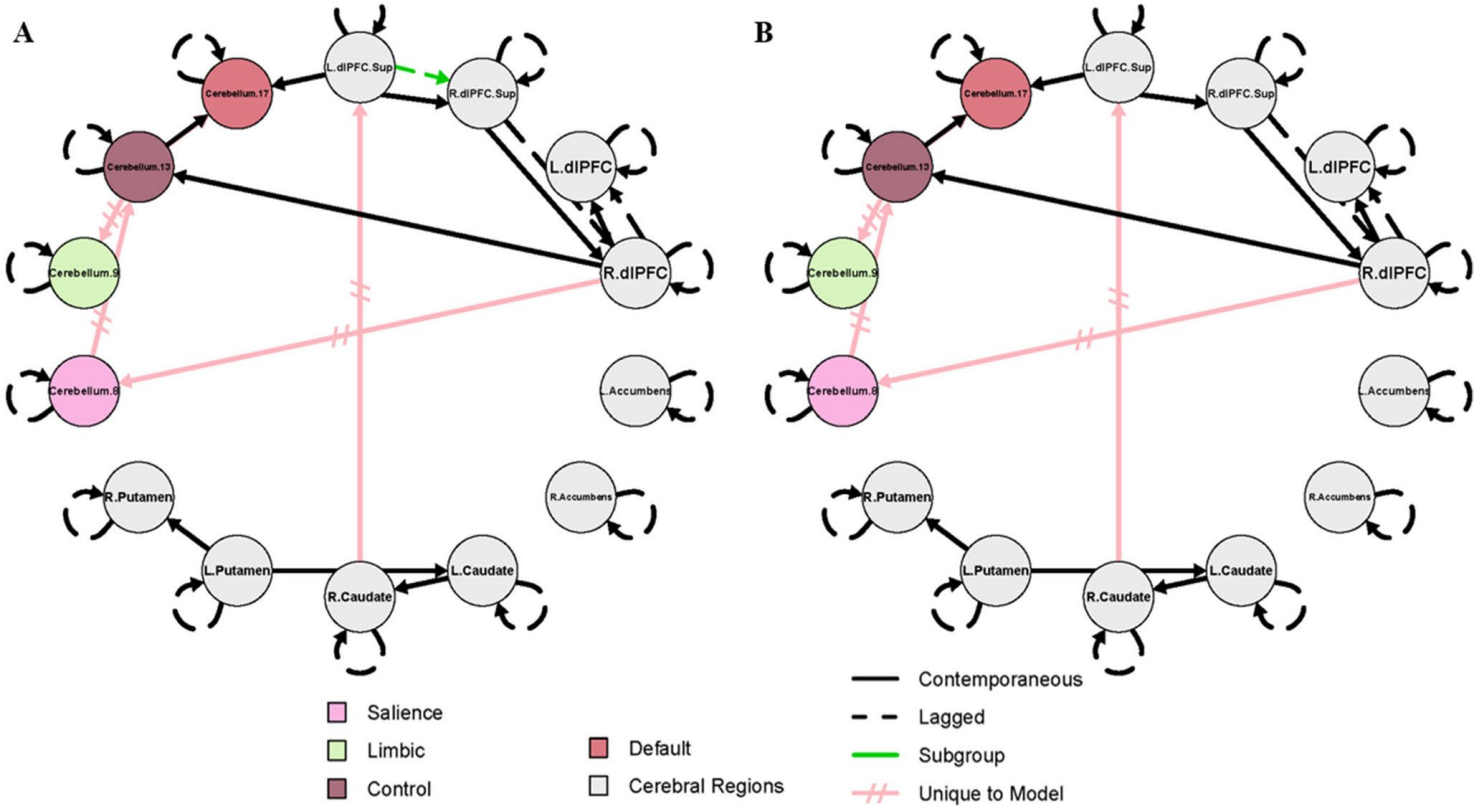
Effective connectivity maps for individuals with schizophrenia with low and high negative symptom severity

The results for the combined SZ + HC model and the SZ-only model are shown in Figs. 1 and 2, respectively. These models were designed to complement one another, and the results should not be interpreted independently. For both figures, group-level paths (present across 75% of the full sample) that appear identical in both SZ + HC and SZ-only models, are represented by black lines. Group-level paths unique to each model are represented by pink, hatched lines. Subgroup-level paths, defined as present in 75% or more of either subgroup, appear in green. The color scheme identified for each cerebellar subregion corresponds to the conventions used in the Buckner 17-network atlas.

The left superior dlPFC to right superior dlPFC, right superior dlPFC to right dlPFC, right dlPFC to left dlPFC, left putamen to right putamen, left putamen to left caudate, left caudate to right caudate, and left superior dlPFC to cerebellum 17 contemporaneous (simultaneous) paths were present in both the SZ + HC and SZ-only models. In addition, a lagged (across-time at a lag of one), group-level path from right dlPFC to left dlPFC was observed in both models.

Several paths were unique to the model that contains both HC and SZ and do not appear in the SZ-only models, including left caudate to left superior dlPFC, cerebellum 17 to cerebellum 13, cerebellum 13 to cerebellum 8, cerebellum 8 to cerebellum 9. In the subgroups of the SZ + HC model, all subgroup-level paths were exclusive to the SZ subgroup and absent in HCs. These include a lagged path from the right superior dlPFC to the right dlPFC, and contemporaneous paths from the right dlPFC to cerebellum 13 and then from cerebellum 13 to cerebellum 17 (Fig. 1).

Because SZ-only models stratified by PANSS positive and negative subscale severity yielded identical connectivity structures, results are summarized in a single figure (Fig. 2). Symptom-specific differences in path coefficients are reported in **Supplemental Table ****3**. In addition to the group-level paths that appeared in both models, as described above, the following contemporaneous connections were specific to the SZ-only models: right dlPFC to cerebellum 8, cerebellum 8 to cerebellum 13, cerebellum 13 to both cerebellum 9 and 17, and right caudate to left superior dlPFC. A lagged subgroup-level path from the left superior dlPFC to right superior dlPFC was specific to the low subgroup in each SZ-only model (i.e. low positive or low negative). Because no subgroup-level path appeared exclusive to the HC subgroup in the SZ + HC model (i.e. no connectivity path appeared in at least 75% of HC without also being relevant to SZ), a HC-only model was not tested. Of note, the bilateral nucleus accumbens did not significantly contribute to any model. 

Effective connectivity map for schizophrenia patients with **A**) low negative symptom severity (PANSS negative score below 14; *n* = 31); **B**) high negative symptom severity (PANSS negative score of 14 or greater; *n* = 24). Identical group- and subgroup-level connectivity structures were observed when stratified by positive or negative symptom severity. Black lines indicate group-level parameters, relevant to 75% of participants in the full sample shared across SZ + HC models and SZ-only models. Pink, hatched lines represent group-level parameters absent in the SZ + HC model. Green lines indicate parameters relevant to 75% of participants within the subgroup. The color scheme for each cerebellar subregion corresponds to the conventions used in the Buckner 17-network atlas. Lagged paths (R.dlPFC.Sup to R.dlPFC, R.dlPFC to L.dlPFC, and L.dlPFC.Sup to R.dlPFC.Sup) are constrained to a lag of 1. L.dlPFC.Sup to Cerebellum.17 (contemporaneous; uncorrected *p* = 0.010) high < low negative symptom severity. L./R.dlPFC = Left/Right dorsolateral prefrontal cortex; L./R.dlPFC.Sup = Left/Right superior dorsolateral prefrontal cortex; Cerebellum regions = anatomical coordinates - corresponding cortical network: Cerebellum.8 = Crus I-II, VI, VIIb, - Salience; Cerebellum.9 = Lobule VIII, Vermis III - Limbic; Cerebellum.13 = Crus I, VIIb - Control; Cerebellum.17 = Crus I-II - Default Mode.

This two-step analytic approach clarified the prevalence of specific connectivity paths in schizophrenia. In the combined SZ + HC model, several paths (right superior dlPFC to dlPFC, dlPFC to cerebellum 13, and cerebellum 13 to 17) were present in at least 75% of SZ participants (i.e. subgroup-level) but not prevalent enough across the full sample to meet the group-level threshold. However, in the SZ-only models, these same paths emerged at the group level, indicating presence in at least 75% of SZ participants. Thus, the combined model established that these paths exist in a majority of individuals with SZ, and the SZ-only model further confirmed their robustness across symptom severity.

### Group Comparisons

When subgroup category (i.e. HC or SZ, low or high symptom severity) was used as a predictor, several group-level paths were of significant interest in both models.

In the SZ + HC model, contemporaneous paths from cerebellum 13 to cerebellum 8 (uncorrected *p* = 0.01), cerebellum 8 to cerebellum 9 (uncorrected *p* = 0.025), and two autoregressive paths in the left putamen (uncorrected *p* = 0.038), and the right caudate (uncorrected *p* = 0.046) had weaker effects in SZ compared to HC.

In the negative symptom severity model, a contemporaneous path from the left superior dlPFC to cerebellum 17 had higher values in the high compared to the low negative symptom severity subgroup (uncorrected *p* = 0.010).

In the positive symptom severity model, autoregressive paths showed lower values in the right superior dlPFC (uncorrected *p* = 0.016) and higher values in cerebellum 9 (uncorrected *p* = 0.046) in the high compared to the low positive symptom subgroup.

Moreover, several covariates had significant relationships with path coefficients even after FDR correction. Across models, age was significantly (negatively) associated with the left putamen to the right putamen (*p* = 0.001), as well as an autoregressive path in the left superior dlPFC (*p* = 0.001). In the SZ + HC model, the autoregressive path in the right dlPFC was also negatively associated with age (*p* = 0.040). Within the negative model, age was also negatively associated with the contemporaneous left putamen to right putamen connection (*p* = 0.041) and the autoregressive left superior dlPFC (*p* = 0.005). In the positive symptom model, age was negatively associated with the left superior dlPFC to cerebellum 17 (*p* = 0.027), the left putamen to right putamen (*p* = 0.027), and an autoregressive path in the left superior dlPFC (*p* = 0.011); for details, see **Supplemental Table ****3**.

## Discussion

The present study investigated alterations in effective connectivity among cortico-striato-cerebellar networks in individuals with SZ compared to HCs. A data-driven approach using metrics from ALFF/fALFF revealed global and regional fluctuations that informed the selection of key cerebellar nodes. These measures, while informative on their own, were complemented by their inclusion in effective connectivity modeling to understand directional influences within cortico-striato-cerebellar circuitry. By integrating ALFF/fALFF with effective connectivity analysis, we identified multiple pathways that differentiate not only individuals with SZ from healthy individuals, but also SZ subgroups with higher or lower symptom severity based on median split. In individuals with SZ, we observed additional contemporaneous paths between cerebellar nodes and between the cerebellum and PFC, as well as an additional lagged path in the PFC that were not present in healthy controls. These findings suggest that abnormal intrinsic activity in the cerebellum may contribute to broader disruptions in information flow across prefrontal-cerebellar circuits in schizophrenia.

Cerebellar subregions selected for the GIMME analysis appeared in the lateral posterior and medial anterior cerebellum. Specifically, according to the Buckner atlas, cerebellum 8 includes voxels within lobules VI and VIIb, and crus I-II; cerebellum 9 is largely lobule VIIIb; cerebellum 13 also is comprised of parts of crus I and lobules VIIb; and cerebellum 17 includes voxels within crus I-II. Additionally, cerebellum 9 spreads into the anterior cerebellum, specifically vermis III. However, these parcellations do not precisely correspond to anatomical boundaries and do not encompass the full extent of these regions. We note that the initial ALFF/fALFF analyses were conducted voxel-wise rather than at the level of predefined Buckner-network ROIs to avoid presupposing network involvement and preserve sensitivity to spatially focal abnormalities within the cerebellum. Because the Buckner networks span large and functionally heterogeneous territories, averaging intrinsic activity across an entire network may obscure localized effects. Accordingly, network labels were used to contextualize clusters rather than define ROIs a priori.

Although previous studies have reported lower ALFF in the vermis and posterior cerebellum in individuals with schizophrenia compared to healthy controls, few have directly investigated how these alterations relate to clinical symptoms [62, 63]. One such study found a negative correlation between language symptoms and ALFF in the posterior lobe, suggesting that intrinsic cerebellar activity may bear some relevance to symptom expression [64]. It is important to note that the posterior cerebellum encompasses several functionally distinct subregions. Our identified cerebellar nodes reflect this anatomical and functional heterogeneity. By using an integrative framework, our study contributes a system-level perspective that complements prior work focused on localized signal differences.

Effective connectivity modeling using GIMME identified group differences in connectivity in several paths between healthy controls and individuals with schizophrenia, although none of these differences survived correction for multiple comparisons. This suggests that broad group effects may be subtle or obscured by within-group variability. However, model-specific pathways, that is, group-level paths identified within a specific model (e.g., the combined SZ + HC, or SZ-only sample), revealed distinct connectivity patterns, particularly those involving the cerebellum, that may be informative to the pathophysiology of schizophrenia. In particular, the model that included both healthy controls and individuals with schizophrenia showed group-level paths from cerebellum 13 to 8, and from 8 to 9, suggesting a directional flow of activity between the control and limbic cerebellar representations being mediated by the salience representation. These group-level paths, by GIMME’s criteria, are those shared by 75% of participants in the model sample. Their presence in the combined model may reflect shared, relatively preserved connectivity patterns that are common to a broader population. Conversely, their absence in the SZ-only models means they are not sufficiently present within the SZ group, indicating greater heterogeneity.

In contrast, models that consisted only of individuals with schizophrenia revealed a distinct configuration of cerebellar connectivity. Specifically, new paths appeared from cerebellum 8 to 13, with cerebellum 13 serving as a hub to both cerebellum 9 and 17. This reversed directionality and expanded role of cerebellum 13 suggests altered network organization in schizophrenia, potentially reflecting a shift in how salience-related regions integrate or redistribute information across cerebellar networks. In schizophrenia, the salience network may exert greater influence over control regions, which in turn coordinate with limbic and default mode systems, potentially reflecting disrupted top-down regulatory processes. The involvement of cerebellum 17, associated with the default mode network, further suggests aberrant integration of internally directed thought processes. Together, these connectivity patterns reveal disease-specific network configurations, highlighting cerebellum 13’s central role in coordinating interactions across multiple functional domains.

Notably, several of these schizophrenia-specific paths involved apparent reversals in canonical cerebellar-cortical directionality. Although cerebellar output pathways are classically characterized as feedforward via dentate-thalamo-cortical projections, the prominent cortico-cerebellar and reversed directional influences observed in the schizophrenia-only models reflect altered functional dynamics within closed-loop cerebro-cerebellar circuits. Effective connectivity estimates statistical dependencies over time, rather than direct axonal transmission. In schizophrenia, increased prefrontal influence on cerebellar nodes may reflect maladaptive top-down control, impaired predictive signaling, or compensatory engagement of cerebellar networks in response to cortical dysfunction. Thus, the observed directionality likely indexes dysregulated bidirectional communication within cerebro-cerebellar loops rather than a true reversal of anatomical information flow.

Building on this altered cerebellar network organization, further distinctive patterns emerged in the cortico-striatal-cerebellar circuitry specific to schizophrenia. In contrast to the HC + SZ model, the connection to the left superior dlPFC is driven by the right caudate in this model, as opposed to the left caudate. Finally, in schizophrenia a unique path connects the right dlPFC to cerebellum 8. These findings suggest that within the cerebellum, salience-related regions (cerebellum 8) may drive activity in the cerebellar control-related regions (cerebellum 13), which in turn, influences both the limbic (cerebellum 9) and default (cerebellum 17) regions. This clearly identifies an altered hierarchy of cerebellar activity specific to schizophrenia and emphasizes the importance of exploring within-group differences given the heterogeneity of the disease.

Subgroup-level paths further reinforced these schizophrenia-specific features. In the combined SZ + HC model, the subgroup-level paths (a lagged path from the right superior dlPFC to right dlPFC, and contemporaneous paths from the right dlPFC to cerebellum 13, and cerebellum 13 to 17) appeared exclusively in the SZ subgroup, and were not present in the HC subgroup, indicating that these paths were present in at least 75% of the individuals with SZ. When the model was run with people with schizophrenia alone, using symptom severity to define low and high subgroups, these paths formed the foundation for the group-level paths, emerging as relevant in at least 75% of SZ participants regardless of high or low symptom severity subgroups. This consistency across symptom-defined subgroups suggests that the identified paths are not tied to symptom severity per se but rather represent core features of schizophrenia-related network architecture.

Although group membership (HC or SZ, low or high symptom severity) was not a significant predictor of any path coefficient after FDR correction, several important patterns emerged that suggest differential network dynamics associated with symptom severity. The cerebellar connections unique to the SZ + HC model were correlated with group at a marginal level, as were autoregressive estimates for the left putamen and right caudate. In all instances, effective connectivity was diminished in individuals with schizophrenia relative to healthy controls. Lower effective connectivity between these cerebellar subregions (cerebellum 13 to 8, and 8 to 9) may imply larger network dysfunction, particularly impaired salience-driven engagement of the control network and cognitive modulation of affect. This interpretation is supported even more by the finding that these group-level connections were absent from the SZ-only model. Additionally, lower temporal autocorrelation in the striatum as seen here could be indicative of dopamine-related disruptions that contribute to impairments in reward processing and goal-directed behaviors commonly seen in schizophrenia. **Supplemental Fig. ****3** shows the distribution of significant predicted effective connectivity values in participants with schizophrenia and healthy comparisons.

Within the SZ-only model, examining differences in by positive symptom severity, the autoregressive estimate of cerebellum 9, a region associated with sensorimotor and limbic processes, was negatively associated with symptom severity, suggesting a reduction in self-inhibition in this region as symptoms intensify. In contrast, the autoregressive estimate of the right superior dlPFC showed a positive association, indicating increased self-inhibition with more severe positive symptoms. This may reflect a compensatory mechanism whereby frontal control systems attempt to regulate or suppress disorganized thoughts. While these findings did not survive correction, they provide preliminary evidence that positive symptom severity may be linked to altered self-regulatory dynamics in both cerebellar and prefrontal systems. **Supplemental Fig. ****4** illustrates the distribution of predicted effective connectivity values for the regions described above in schizophrenia participants by positive and negative symptom severity.

Interestingly, the SZ-only model, examining differences in negative symptom severity, showed distinct patterns of connectivity with only a contemporaneous path from the left superior dlPFC to cerebellum 17 being negatively associated with symptom severity. Cerebellum 17 includes voxels in Crus I and II, regions that are reciprocally connected with the prefrontal cortex, with projections from the dlPFC to Crus I and II returning to the dlPFC via the dentate nucleus. This circuit has been tied to motor, cognitive, and executive control symptoms related to a variety of diseases, including Parkinson’s Disease and obsessive-compulsive disorder (Caspers et al., 2017; H. Li et al., 2020). However, similar connectivity patterns have not been previously reported in schizophrenia to our knowledge. These results imply that within-region temporal autocorrelation dynamics may be more sensitive to negative symptom severity (see **Supplemental Table ****3** for full model estimates).

While most group differences in path coefficients in the secondary analyses did not survive multiple comparison correction, several connectivity patterns were significantly associated with age, suggesting aging-related processes may confound diagnostic comparisons. These were observed in both inter-regional (e.g. left putamen to right putamen) and autoregressive (e.g. left superior dlPFC at a lag) paths, indicating that age influences both within- and between-region effective connectivity. This aligns with prior research showing age-related declines in rs-functional connectivity in prefrontal and subcortical networks often seen in schizophrenia. Our effective connectivity findings add to this, suggesting that both synchrony and direction of information flow shifts with age in schizophrenia. Stratifying the schizophrenia group by symptom severity revealed more nuanced effects: although connectivity maps were structurally similar across subgroups defined by symptom severity, the secondary statistical analyses identified distinct predictors of connectivity strength that reached significance at the uncorrected level. In the positive symptom model, age was negatively associated with connectivity strength in cerebellar-prefrontal and striatal-striatal pathways, indicating that these connections were stronger among younger individuals with more prominent positive symptoms. This pattern of stronger cerebellar-prefrontal and intra-striatal connectivity in younger individuals could reflect a transient phase of hyperconnectivity associated with active symptom presentation in the earlier stages of the disease.

To assess whether these effective connectivity findings offer additional insights beyond traditional methods, we compared them with static functional connectivity measures derived from the same sample and set or ROIs (see **Supplement [**Figs. 1 and 2**]** for detailed methodology and full results). Effective connectivity revealed several unique interactions that were not identified as significant by static connectivity methods, even with a generous significance threshold (uncorrected for multiple testing). Static connectivity was unable to detect connections identified by effective connectivity within the SZ + HC model, which included links between the left caudate and left superior dlPFC, as well as two paths specific to the SZ subgroup within this model (the right superior dlPFC and the right dlPFC and right dlPFC and cerebellum 13). Within the SZ-only model, all intra-cerebellar connections were unique to the effective connectivity models and went undetected by static connectivity methods. This is also true for the right dlPFC and cerebellum 8 connection.

Conversely, several connections were identified by both effective and static connectivity methods. Among the paths that were unique to the SZ + HC model, connections between cerebellar pairs 13 and 8, and 8 and 9, showed both reduced effective and static connectivity in individuals with SZ relative to HC. Among the paths that appeared at the group-level in both SZ + HC and SZ-only models, including the left putamen and left caudate, the left and right putamen, the left and right superior dlPFC, and cerebellum 13 and 17, all were represented by reduced static connectivity in SZ relative to HC. However, these paths were not flagged as group-differentiating in the effective connectivity models because they met the group-level inclusion and were therefore common to both groups. Finally, within the SZ-only models, a unique group-level path of the right caudate and left superior dlPFC was not associated with group in effective connectivity models but was associated with positive symptom predominance in the static connectivity analysis, indicating that this while this connection is commonly present across individuals with schizophrenia, its strength, rather than directional influence, may vary with symptom severity.

These findings support the cerebellum’s role as a modulatory hub within cognitive-affective circuits implicated in schizophrenia. Moreover, they highlight the added value of effective connectivity in detecting directional, dynamic relationships that static approaches may overlook. In particular, effective connectivity may be more sensitive to intra-cerebellar connections, especially in clinically heterogeneous populations. By clarifying directional influences of cerebellar outputs on cortical targets, this approach contributes to a more precise model of how cerebellar dysfunction may drive network-level disruptions and contribute to core schizophrenia symptoms.

## Conclusions

Our findings provide novel insights into the network-level disruptions in schizophrenia, particularly involving the cerebellum’s shifting role in mediating control, salience, and limbic networks. Effective connectivity modeling revealed dynamic relationships that extend beyond traditional static functional connectivity measures, highlighting both the sensitivity of GIMME and the importance of individualized patterns over group averages. Although broad group differences rarely reached statistical significance, the emergence of stable cerebellar alterations across schizophrenia models suggests a biologically meaningful reorganization of intra-cerebellar and cerebro-cerebellar communication. Although symptom severity was operationalized categorically in the present study, the observed variability in path coefficients across symptom-defined subgroups suggests that future work examining symptom severity along a continuum may provide additional insight.

### Limitations and Opportunities

Several subjects had to be excluded due to missing data in the cortical and cerebellar regions of interest. While the number of subjects included was still substantial following strict exclusionary criteria, results could be bolstered by repeating the analysis on a larger dataset. The use of voxel-based ALFF/fALFF to define ROIs may inadvertently localize clusters that do not span entire networks. Future work could directly compare voxel-wise and network-averaged approaches to assess how analytic scale influences detection of cerebellar abnormalities in schizophrenia. Additionally, group-level ALFF/fALFF may overlook individual variability in network organization. Future studies could extend current findings by directly examining effective connectivity patterns using targeted anatomical parcellations. While GIMME offers a powerful approach for estimating directional interactions, it infers causality from statistical patterns rather than direct physiological measures. Incorporating multimodal imaging approaches could enhance mechanistic insight. To better characterize the role of autoregressive and contemporaneous dynamics in symptom expression, future studies using targeted datasets with greater within-group homogeneity may improve sensitivity to symptom-specific connectivity patterns and address the current limitations related to statistical power.

While cerebello-cerebral circuits are frequently examined in relation to cognitive domains such as executive function and working memory [65], the present study focused specifically on positive and negative symptom dimensions. This focus was motivated by their clinical relevance, particularly the persistence and treatment resistance of negative symptoms, which remain a major unmet need in schizophrenia research. Future work could extend the present framework to examine how cerebellar dysconnectivity contributes to cognitive impairments, allowing for a more comprehensive integration of cerebro-cerebellar networks across multiple symptom dimensions. Moreover, participants with schizophrenia in the COBRE dataset were medicated, raising the possibility that some connectivity differences reflect treatment effects rather than disease-related changes. Antipsychotic dose (converted to olanzapine equivalents) was included as a covariate in secondary regression models to address this concern; however statistical control cannot fully account for the complex and potentially cumulative effects of long-term medication exposure. Future work could address this by examining medication-naïve or first-episode cohorts. Finally, transdiagnostic comparisons may help contextualize cerebellar dysconnectivity within broader frameworks of brain function and dysfunction.

## Supplementary Information

Below is the link to the electronic supplementary material.

Supplementary Material 1

## Data Availability

Imaging, assessment, and demographic data that support the findings of this study (obtained from Centers of Biomedical Research Excellence, Phase I study from the Mind Research Network in Albuquerque, NM) can be accessed at the following link: https://coins.trendscenter.org/#exchangepost-processed time series data, cleaned demographic and assessment data, and associated code can be accessed here: https://osf.io/g3bkf/?view_only=e7b73749a45c4b69940e68c642254533.
