# Supplementary material for: Effective Connectivity Identifies Divergent Cerebro-Cerebellar Network Organization in Schizophrenia
Source: Cerebellum. 2026 Mar 23;25(2):38. doi: 10.1007/s12311-026-01983-7 (PMC13006459; doi:10.1007/s12311-026-01983-7)
Supplement: Supplementary file 1 — Supplementary Material 1 [file 12311_2026_1983_MOESM1_ESM.docx]

**Supplementary Table 1**. Cortical parcels corresponding to selected brain ROIs

| **Cortical Parcel** | |
| --- | --- |
| **L dPFC** | **R dPFC** |
| *17 Networks_LH_ContA_PFCd_1* | *17 Networks_RH_ContB_PFCld_1* |
| *17 Networks_LH_ContB_PFCd_1* | *17 Networks_RH_ ContB _PFCld_2* |
|  | *17 Networks_RH_ ContB _PFCld_3* |
| **L dPFC Sup** | **R dPFC Sup** |
| *17 Networks_LH_DefaultB_PFCd_2* | *17 Networks_RH_DefaultA_PFCd_1* |
| *17 Networks_LH_DefaultB_PFCd_3* | *17 Networks_RH_DefaultA_PFCd_2* |
| *17 Networks_LH_DefaultB_PFCd_4* | *17 Networks_RH_DefaultB_PFCd_2* |
|  |  |
|  |  |

*ROI* = region of interest; *L* = left; *R* = right; *dPFC* = dorsal prefrontal cortex; *Sup* = Superior.

***Effective Connectivity Statistical Analysis—Path Presence***

**Methods**

To determine whether the presence of a specific subgroup-level path within the models were associated with group, we conducted a chi-square test comparing the frequency of the path across subgroups. A logistic regression was then performed for each subgroup-level path generated across the three models (HC vs. SZ: *paths = 3*; negative symptom severity: *paths = 1*; positive symptom severity: *paths =1*) among participants in whom the path was present, to assess whether subgroup membership (i.e. HC vs. SZ or mild vs. severe) predicted path presence while accounting for potential confounding variables (age, sex, and mean framewise displacement, illness-to-life ratio, and Olanzapine equivalency scores).

**Results**

There was no significant difference in the frequency of occurrence of the subgroup-level paths between groups in the HC vs. SZ. Subgroup membership was not a significant predictor of path presence for any of the paths tested, with the exception of the model defined by positive symptom severity, which showed a significant association (OR = -2.576, *p* = 0.015).

**Supplementary Table 2:** Summary of symptom and group effects on path presence and strength

| **Path** | **Test Type** | **Variable** | **Estimate** | **p-value** |
| --- | --- | --- | --- | --- |
| *RH dlPFC superior → RH dlPFC lagged* | Presence (GLM) | Group (SZ vs HC) | OR = -0.226 | 0.556 |
|  | Strength (r) | Positive Symptoms | r = 0.160 | 0.358 |
|  | Strength (r) | Negative Symptoms | r = 0.022 | 0.901 |
|  | Strength (r) | Composite Score | r = 0.102 | 0.560 |
| *RH dlPFC → Cerebellum 13* | Presence (GLM) | Group (SZ vs HC) | OR = -0.472 | 0.231 |
|  | Strength (r) | Positive Symptoms | r = -0.103 | 0.557 |
|  | Strength (r) | Negative Symptoms | r = 0.002 | 0.992 |
|  | Strength (r) | Composite Score | r = -0.079 | 0.653 |
| *Cerebellum 13 → Cerebellum 17* | Presence (GLM) | Group (SZ vs HC) | OR = 0.072 | 0.844 |
|  | Strength (r) | Positive Symptoms | r = -0.217 | 0.257 |
|  | Strength (r) | Negative Symptoms | r = -0.195 | 0.312 |
|  | Strength (r) | Composite Score | r = 0.017 | 0.931 |
| *LH dlPFC superior → RH dlPFC superior lagged* | Presence (GLM) | Group (Mild vs. Severe) | OR = -2.576 | 0.015* |
|  | Strength (r) | Positive Symptoms | r = -0.258 | 0.088 |
|  | Strength (r) | Negative Symptoms | r = -0.045 | 0.770 |
|  | Strength (r) | Composite Score | r = -0.159 | 0.296 |

** p >* 0.05

***Static Connectivity***

**Methods**

Static atlas-based connectivity matrices for the Schaefer combined atlas were calculated using HALFpipe. Connectivity values are correlations between BOLD signals of each ROI from the Schaefer combined atlas across a time-series calculated using nilearn’s connectivity measure. The connectivity analysis data was then filtered in R to only include the desired brain regions (bilateral putamen, bilateral caudate, bilateral accumbens area, cerebellum, bilateral dlPFC, and bilateral superior dlPFC). The dlPFC areas of interest consisted of several atlas regions and any correlates from these areas were combined into a single averaged data point, which was used in the regression. Several regressions were run with the PANSS total negative symptoms scores, PANSS total positive symptoms, and PANSS composite scores as predictors and the brain regions of interest as dependent variables. The covariates in all PANSS regressions were age, gender, total Olazapine equivalent dose, and framewise displacement. A group-level comparison regression was also completed in R between healthy controls and individuals with schizophrenia. In this regression, group is a predictor, brain regions of interest are dependent variables, and age, gender, and framewise displacement are covariates. BH-corrected p-values were added to results for all regressions to account for multiple comparisons. The results are presented in the figures below: Supplemental Figure 1 shows the connectivity matrices filtered at an uncorrected *p* < .05, reflecting reduced connectivity in SZ relative to HC, on the left. An exception was the correlation between left DLPFC and cerebellum network 8 where SZ > HC. The heatmap on the right shows the general pattern of increased connectivity related to increased symptom scores.

**Supplementary Figure 1:** Heatmaps representing ROI × ROI static connectivity of regression estimates


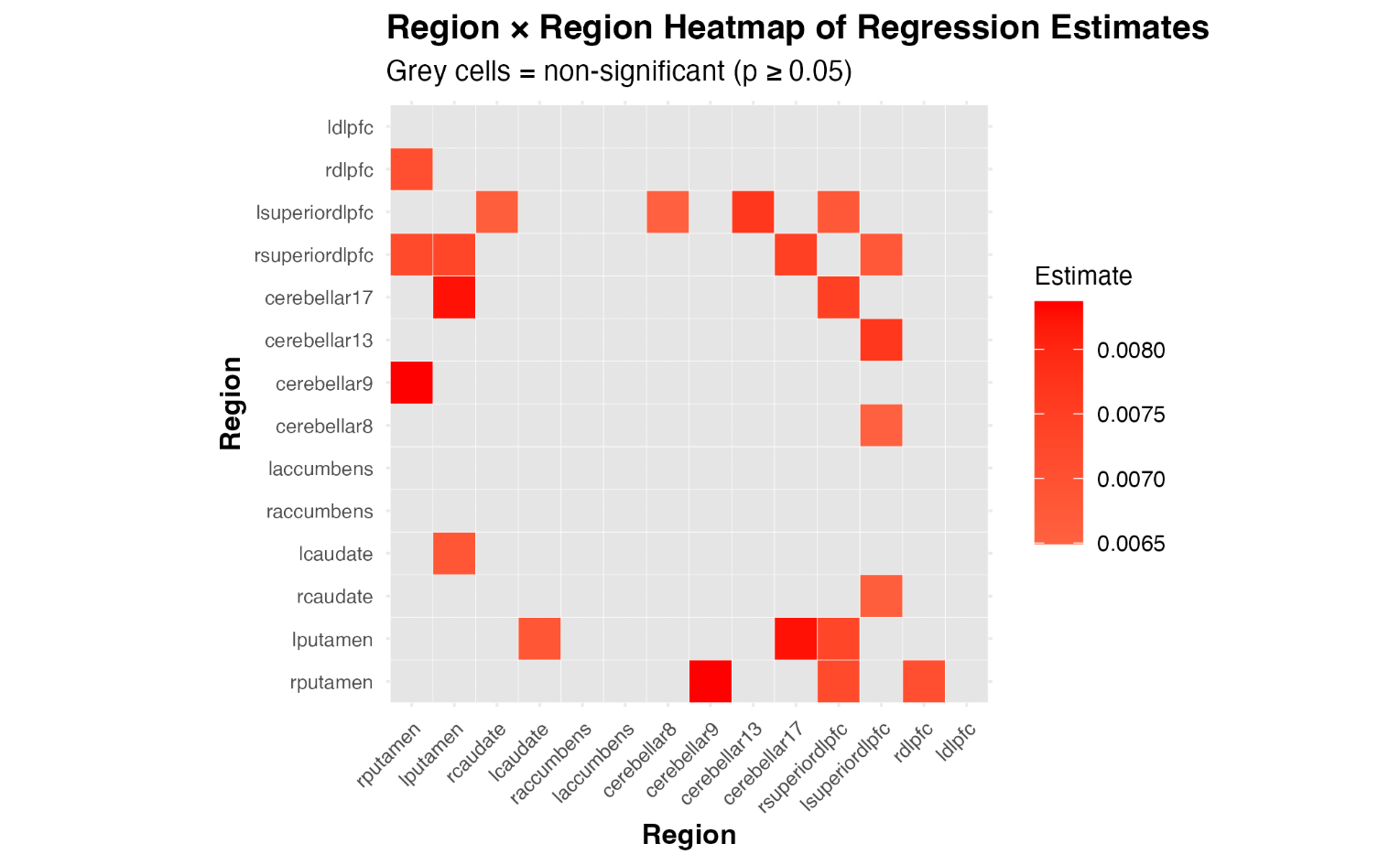

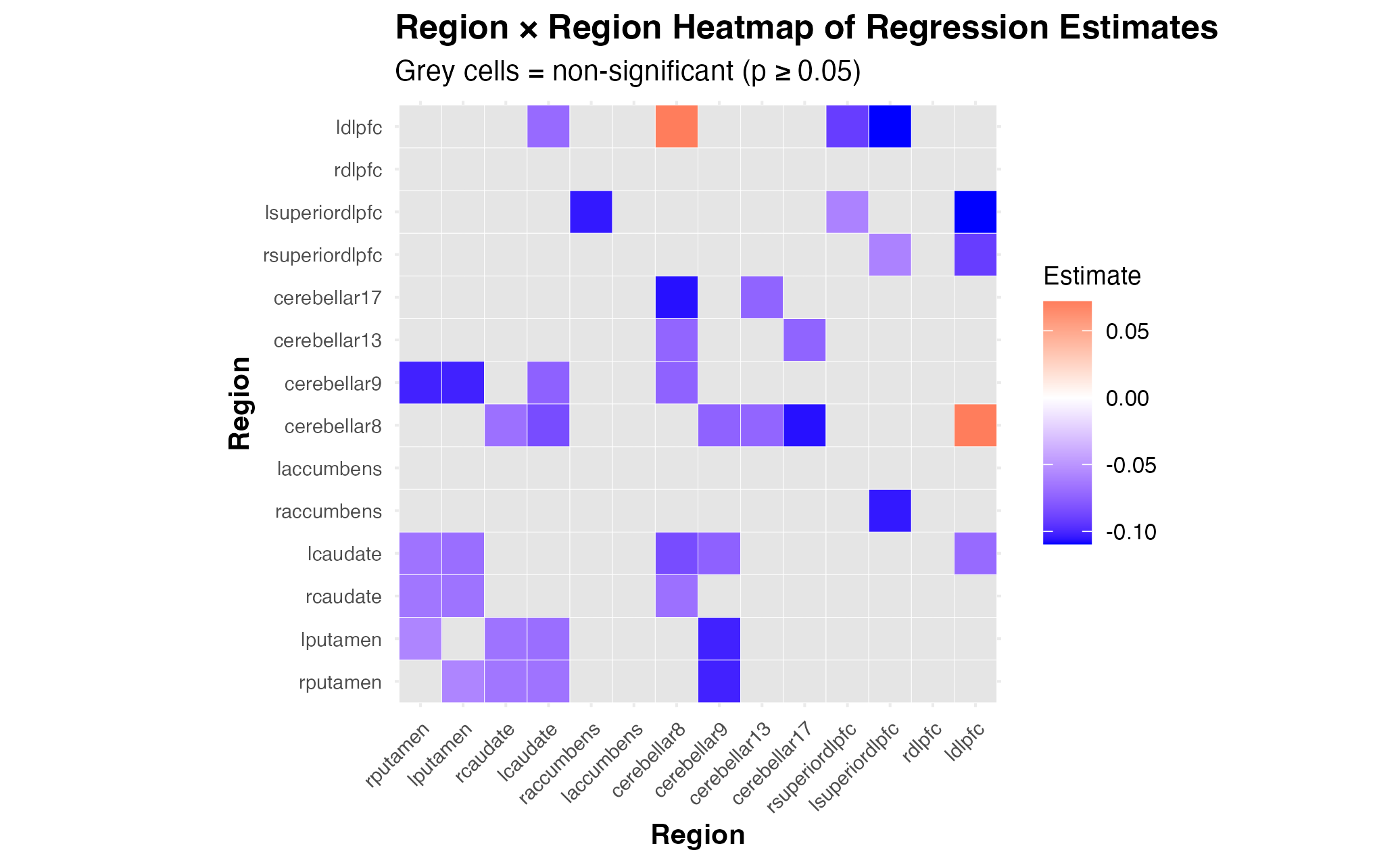


**A**

**B**

Heatmaps representing ROI × ROI static connectivity of regression estimates for **A)** healthy control vs. individuals with schizophrenia, with blue indicating hypoconnectivity in SZ relative to HC and **B)** individuals with schizophrenia only, with brighter red indicating a higher composite PANSS score, or positive symptom predominance. *Right Putamen × Left Putamen (HC vs. SZ)*: Bonferroni corrected p* = 0.0246. All other static connections did not pass Bonferroni correction (uncorrected *p* ≤ 0.05).

**Supplemental Figure 2:** Regression-based static connectivity patterns in healthy controls and individuals with schizophrenia


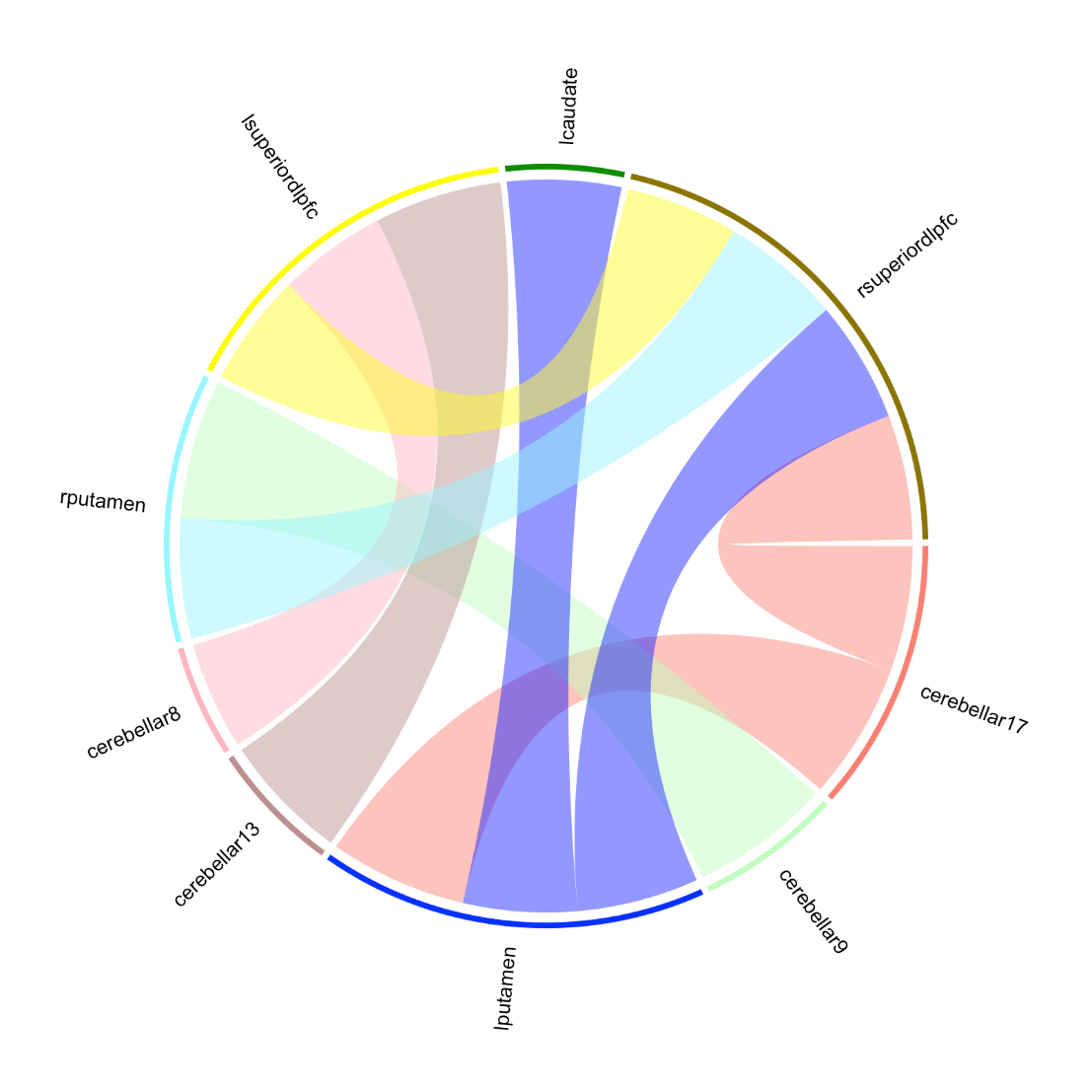

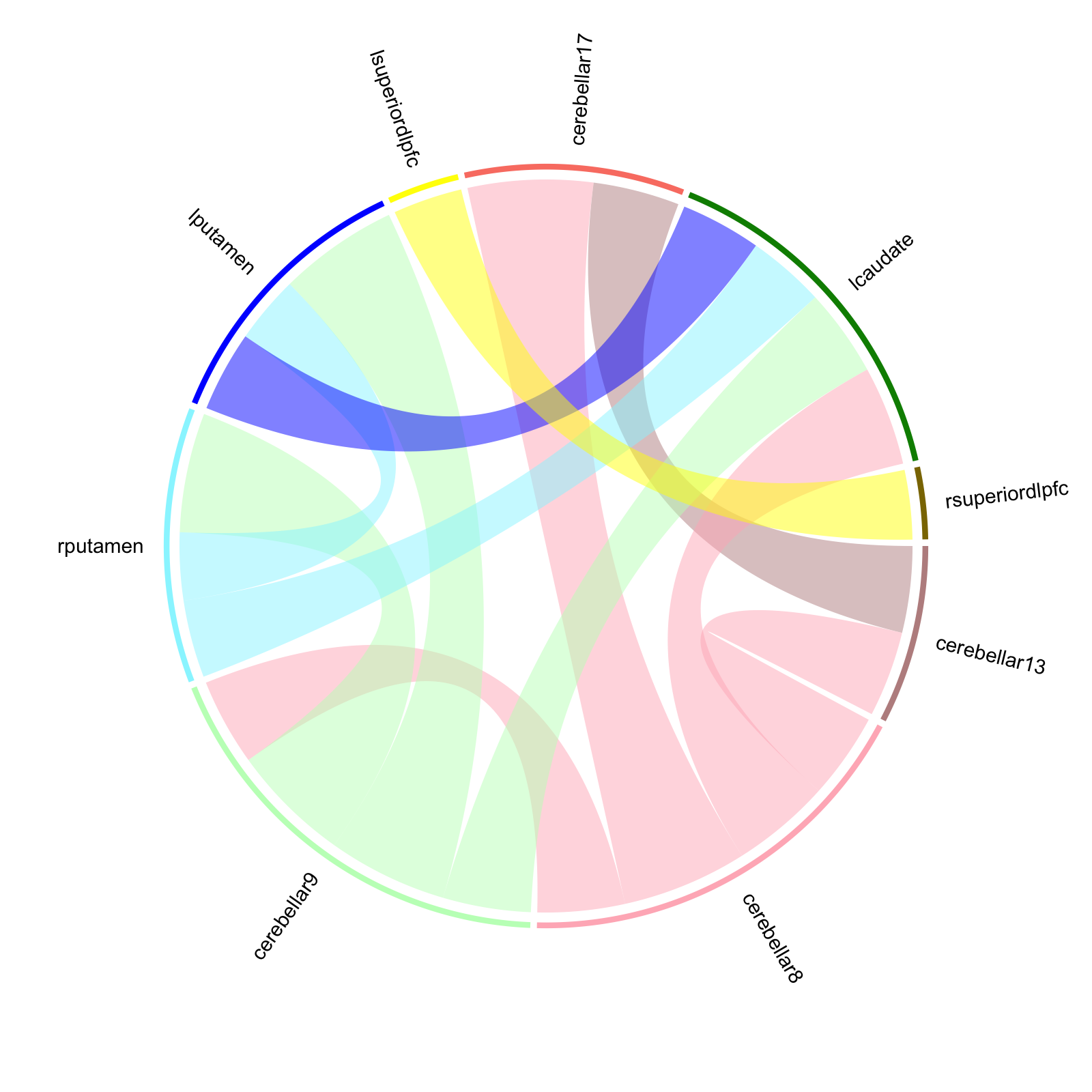


**A**

**B**

Significant connections at the uncorrected *p* < 0.05 level between regions included in the GIMME models (bilateral putamen, left caudate, bilateral superior dlPFC, cerebellar 8, cerebellar 9, cerebellar 13, cerebellar 17) for **A)** healthy controls vs. schizophrenia and **B)** schizophrenia-only by composite PANSS score. The ROIs included in these graphs are connections that appear in both the static and effective connectivity analyses. The thickness of each cord connecting the regions represents the strength of the regression estimate of the connection.

**Supplemental Table 3:** Group-level path coefficient analysis

| **Path** | **Predictor** | **β coefficient** | **stderr** | **t** | **p** | **CI Lower** | **CI Higher** | **p (FDR)** |
| --- | --- | --- | --- | --- | --- | --- | --- | --- |
| *HC vs SZ* | | | | | | | | |
| Cerebellum 13 🡪 Cerebellum 8 | Group | -0.137 | 0.053 | -2.612 | 0.010 | -0.241 | -0.033 | 0.261 |
| Cerebellum 8 🡪 Cerebellum 9 | Group | -0.118 | 0.052 | -2.272 | 0.025 | -0.221 | -0.015 | 0.298 |
| Left Putamen 🡪 Left Putamen lag | Group | -0.072 | 0.035 | -2.095 | 0.038 | -0.141 | -0.004 | 0.298 |
| Right Caudate 🡪 Right Caudate lag | Group | -0.039 | 0.019 | -2.016 | 0.046 | -0.077 | -0.001 | 0.298 |
| Left superior dlPFC 🡪Left superior dlPFC lag | Age | -0.005 | 0.001 | -4.241 | 0.000 | -0.008 | -0.003 | 0.001** |
| Right dlPFC 🡪 Right dlPFC lag | Age | -0.004 | 0.001 | -2.885 | 0.005 | -0.007 | -0.001 | 0.040* |
| Left Putamen 🡪 Right Putamen | Age | -0.005 | 0.001 | -4.034 | 0.000 | -0.008 | -0.003 | 0.001** |
| *Negative Symptom Severity* | | | | | | | | |
| Left superior dlPFC 🡪 Cerebellum 17 | Group | 0.106 | 0.039 | 2.691 | 0.010 | 0.027 | 0.186 | 0.283 |
| Left superior dlPFC 🡪Left superior dlPFC lag | Age | -0.009 | 0.002 | -4.056 | 0.000 | -0.013 | -0.005 | 0.005** |
| Left Putamen 🡪 Right Putamen | Age | -0.010 | 0.003 | -3.144 | 0.003 | -0.016 | -0.004 | 0.041* |
| *Positive Symptom Severity* | | | | | | | | |
| Right superior dlPFC 🡪 Right superior dlPFC lag | Group | -0.117 | 0.047 | -2.498 | 0.016 | -0.212 | -0.023 | 0.463 |
| Cerebellum 9 🡪 Cerebellum 9 lag | Group | 0.091 | 0.045 | 2.046 | 0.046 | 0.002 | 0.181 | 0.535 |
| Left superior dlPFC 🡪Left superior dlPFC lag | Age | -0.008 | 0.002 | -3.819 | 0.000 | -0.013 | -0.004 | 0.011* |
| Left superior dlPFC 🡪 Cerebellum 17 | Age | -0.006 | 0.002 | -3.241 | 0.002 | -0.010 | -0.002 | 0.027* |
| Left Putamen 🡪 Right Putamen | Age | -0.010 | 0.003 | -3.153 | 0.003 | -0.016 | -0.004 | 0.027* |
| Left dlPFC 🡪Left dlPFC lag | FD | -0.546 | 0.165 | -3.300 | 0.002 | -0.878 | -0.213 | 0.044* |
| Cerebellum 13 🡪 Cerebellum 17 | FD | 0.804 | 0.268 | 2.999 | 0.004 | 0.265 | 1.342 | 0.044* |
| Cerebellum 8 🡪 Cerebellum 13 | FD | 0.685 | 0.230 | 2.975 | 0.005 | 0.222 | 1.148 | 0.044* |

***** *p >* 0.05; ** *p* > 0.01

**Supplemental Figure 3:** Distribution of Predicted Connectivity Estimates for Healthy Controls and Persons with Schizophrenia


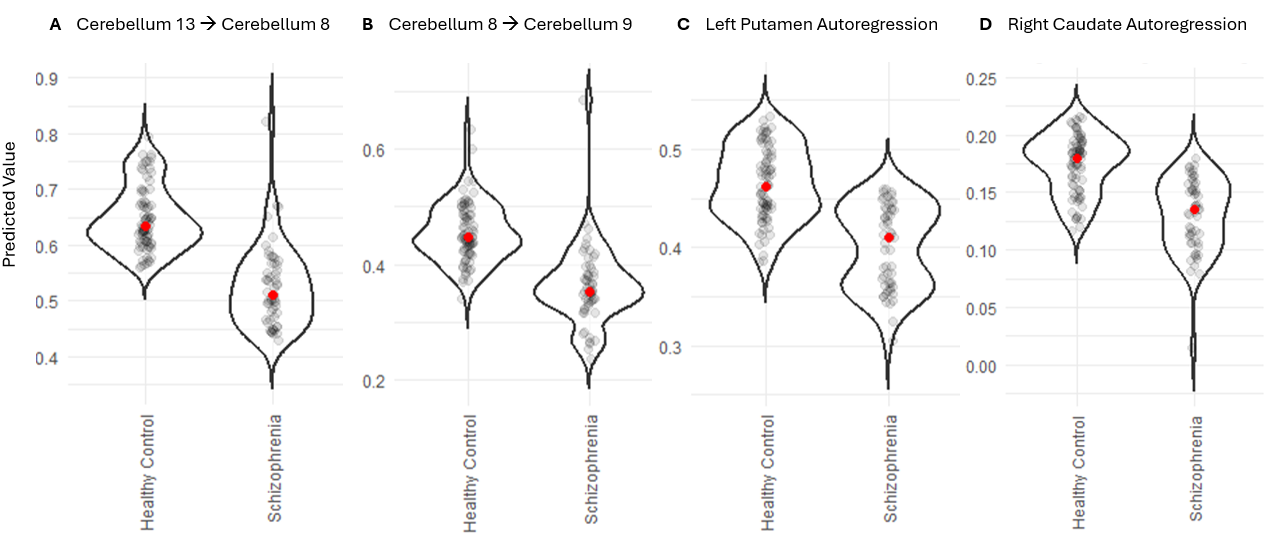


Predicted values of contemporaneous and regional temporal autocorrelation estimates by diagnostic group after controlling for age, gender, and average framewise displacement.

**Supplemental Figure 4:** Distribution of Predicted Connectivity Estimates for Persons with Schizophrenia by Positive and Negative Symptom Severity


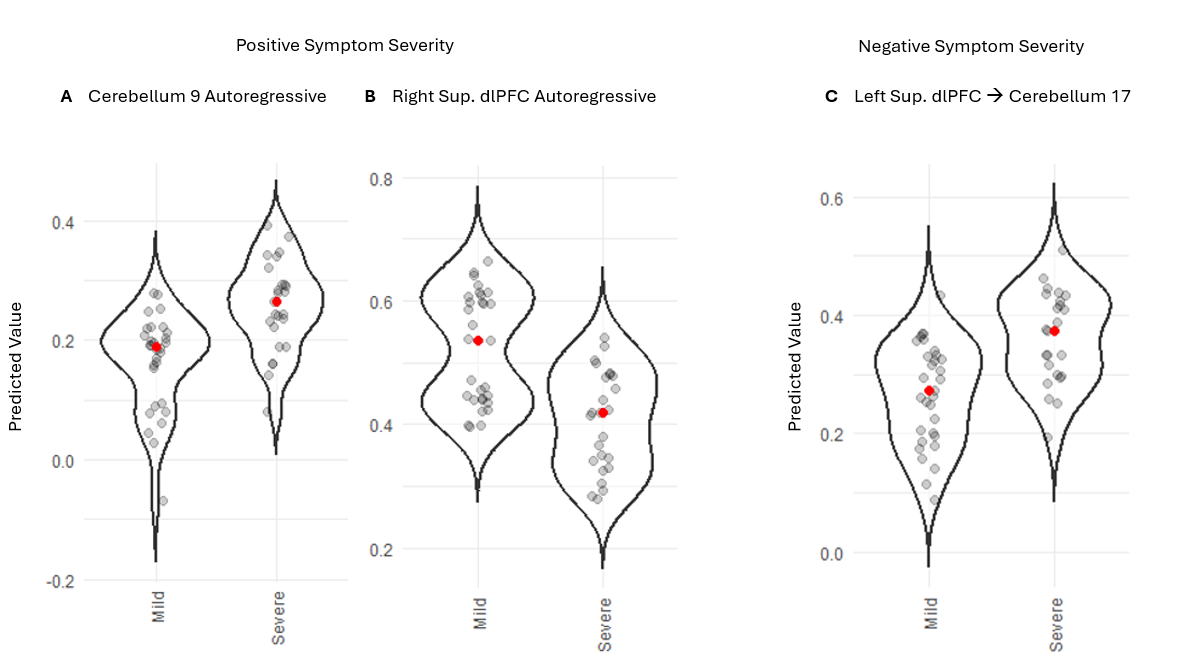


Predicted values of regional temporal autocorrelation estimates by **A, B)** positive symptom severity and contemporaneous values by **C)** negative symptom severity after controlling for age, gender, average framewise displacement, and Olanzapine equivalency scores. *dlPFC* = dorsolateral prefrontal cortex
